# Two Cases of Coma Following the Sudden Retrocession of Ptyalism; One of Which Was Cured by Reproducing the Salivation

**Published:** 1826-09

**Authors:** R. Macleod


### COMA.

*Two Cases of Coma following the sudden Retrocession of Ptyalism; one of which was cured by reproducing the Salivation.* By R. Macleod, M.D.

Some years ago I attended an elderly lady, who became profusely salivated by bluepill, small doses of which had been administered for an hepatic affection. The ptyalism continued, with little change, for eight days, notwithstanding the internal use of diluted sulphuric acid and neutral salts, and the local application of an alum-wash. At the end of this period, and without any obvious cause, the secretion became suddenly diminished, and entirely disappeared in the course of a day. Simultaneously with the suppression of the salivation, she complained of drowsiness: this rapidly passed into coma, and she died on the third day, notwithstanding the use of local and general bleeding, blistering, and purging.

The entire failure of these means in affording any degree of relief, induce me to think that, under similar circumstances, a different method might be employed, with more chance of success. I submit whether the following case does not in some degree justify this idea.

July 19, 1826.—James Bond, aged forty-nine, a painter, residing at No. 4, Bird-in-hand Court, Long Acre, a fortnight ago was attacked with colic, to which he has been occasionally subject for several years. The disease was treated with calomel, in doses of five grains, over-night, followed up by castor-oil next morning, in such quantity as to make the bowels act two or three times each day. Under this plan the colic soon disappeared; but his mouth began to get sore, and at the end of a week he was profusely salivated, blood being occasionally mixed with the saliva. He used an alum gargle, and took neutral salts; notwithstanding which, his mouth continued very sore for several days. Yesterday morning it was observed that the salivation was much diminished, and the patient, thinking himself better, walked out in the evening, for the first time since his illness. He was found soon after, at the end of the court in which he lived, (a distance of only a few yards,) leaning with his back against the wall of a house. On being spoken to, he did not answer, and was carried home in a state of insensibility. Mr. Wade, Apothecary to the Westminster Dispensary, was sent for, who had him cupped on the nape of the neck, to the extent of twelve ounces, and ordered a purgative enema.

*Present state.*—Noon.—He is lying on his back, with a vacant expression of countenance; the mouth is slightly drawn to the left side; the pupils are dilated, but contract on turning his face to the light. He does not answer questions, though put to him in a loud voice, nor does he show any sign of hearing them. When his limbs are pinched, or otherwise incommoded, he moves them. The salivation (which I had observed to be profuse the day before yesterday,) has entirely ceased. Tongue white; bowels acted twice during the night, involuntarily; pulse eighty, full and soft; skin cool; urine passed unconsciously, but, so far as can be ascertained, natural.

Habeat quamprimum Calomelanos gr. x.; et post horas tres Infusi Sennæ ℥ xij. cum Magnesiæ Sulphatis ℥ ij.—Repetatur Haustus secundâ quâque horâ si opus sit.

Evening.—Bowels have been opened three times: no change in the symptoms.

Applicetur Emplast. Lyttæ nuchæ.—Repetatur Haustus Sennæ cras mane.

20th.—Bowels continue open. Blister has not risen. No change in the symptoms.

Repetantur Calomelanos gr. x. quamprimum.—Omittatur Haustus.

21st.—Gums becoming sore, and some saliva runs from the corner of the mouth. Bowels have acted twice to-day. When spoken to, he evidently hears, and seems to understand what is said; but does not speak, although he makes an apparent effort to do so.

Omittantur medicamenta.

22d.—Salivation fully established. Answers, Yes, or No, to questions admitting of a direct reply; makes known his wants by signs; and has taken some tea.

It is unnecessary to continue daily reports of the case, as from this time the patient progressively improved, and all the symptoms gradually disappeared. The salivation was not so profuse as that which preceded the attack of coma: no gargle was used; and it gradually went off in five or six days. The bowels continued to act regularly without medicine;. and he returned to his work the beginning of August, apparently as well as before his illness; but his wife thinks that his mind is not so completely restored as his bodily health.

Should the indication which was here acted upon be adopted by any other practitioner, under similar circumstances, perhaps a more speedy method of restoring the salivation might be preferred,—as by rubbing calomel on the gums, or by fumigation. The circumstance which induced me to give the calomel as above mentioned, was the fact of its having so readily induced ptyalism in the first instance.

*Henrietta-street, Cavendish-square; August* 10, 1826.

